# Quinine—Its Effect upon the Spleen, Liver, &c.

**Published:** 1846-02

**Authors:** 


*Quinine—its effect upon the Spleen, Liver, &c.*—Dr. Van Buren, of the U. S. Army, closes a paper in the N. York Journal of Medicine, on quinine, with the following observations:

"I have never seen a case of disease of the liver or spleen, which could—on the most remote evidence—be attributed to the use of quinine, either in large or small doses.

"Of the qualities of the sulphate of quinine as a *pure tonic in small doses* I have received an unfavorable impression, in the debility following diseases of this climate. Used in this manner I believe that it acts only as a direct and peculiar permanent stimulant, and that it has a greater tendency to induce general irritability of the system than other and more simple vegetable tonics, with a decidedly unpleasant effect in many instances upon the mucous membrane of the intestines, and the brain.

"I have never recognized its action upon the liver.

"After stopping an intermittent fever by a full dose of quinine, unless the system requires decided support, I am not in the habit generally of administering tonics; and I have never witnessed more satisfactory cases of prompt and uninterrupted convalescence than while pursuing this plan of practice. In cases which required a supporting treatment, the indigenous tonics of the country—the prunus Virginiana and cornus Florida—have proved to be valuable medicines.

"According to my observations the unpleasant effects of quinine, in producing ringing in the ears, &c., &c., are more constant and severe in proportion to the smallness of the dose employed. I have seen many patients excessively annoyed by doses of two grains, frequently repeated, who have afterwards, under similar circumstances, taken twenty grains with comparative impunity. The medicine, in solution, has also a greater tendency to produce these symptoms than in pilular form; still the solution is more immediate and efficient in its action.—*N. Y. Jour. Med.*

